# Venous thromboembolic prophylaxis: current practice of surgeons in Australia and New Zealand for major abdominal surgery

**DOI:** 10.1186/s12893-023-02135-y

**Published:** 2023-09-01

**Authors:** Natalie Lott, Tharindu Senanayake, Rosemary Carroll, Jon Gani, Stephen R Smith

**Affiliations:** 1 Surgical Services, John Hunter Hospital, Newcastle, NSW Australia; 2 Hunter Surgical Clinical Research Unit, John Hunter Hospital, New Lambton Heights, Australia; 3 School of Medicine and Public Health, University of Newcastle, Newcastle, NSW Australia; 4 Calvary Mater Hospital, Newcastle, NSW Australia

**Keywords:** Major abdominal surgery, Venous thromboembolism (VTE), Intermittent pneumatic compression devices, VTE risk assessment, Surgeon survey

## Abstract

**Background:**

Surgical prophylaxis for venous thrombo-embolic disease (VTE) includes risk assessment, chemical prophylaxis and mechanical prophylaxis (graduated compression stockings [GCS] ***and/or*** intermittent pneumatic compression devices [IPCD]). Although there is overwhelming evidence for the need and efficacy of VTE prophylaxis in patients at risk, only about a third of those who are at risk of VTE receive appropriate prophylaxis.

**Objective:**

There is debate as to the best combination of VTE prophylaxis following abdominal surgery due to lack of evidence. The aim of this survey was to understand this gap between knowledge and practice.

**Methods:**

In 2019 and 2020, a survey was conducted to investigate the current practice of venous thromboembolism (VTE) prophylaxis for major abdominal surgery, with a focus on colorectal resections. The study received ethics approval and involved distributing an 11-item questionnaire to members of two professional surgical societies: the Colorectal Surgical Society of Australia and New Zealand (CSSANZ) and the General Surgeons Australia (GSA).

**Results:**

From 214 surgeons: 100% use chemical prophylaxis, 68% do not use a risk assessment tool, 27% do not vary practice according to patient risk factors while > 90% use all three forms of VTE prophylaxis at some stage of treatment. Most surgeons do not vary practice between laparoscopic and open colectomy/major abdominal surgery and only 33% prescribe post-discharge chemical prophylaxis. 42% of surgeons surveyed had equipoise for a clinical trial on the use of IPCDs and the vast majority (> 95%) feel that IPCDs should provide at least a 2% improvement in VTE event rate in order to justify their routine use.

**Conclusion:**

Most surgeons in Australia and New Zealand do not use risk assessment tools and use all three forms of prophylaxis regardless. Therfore there is a gap between practice and VTE prophylaxis for the use of mechanical prophylaxis options. Further research is required to determine whether dual modality mechanical prophylaxis is incrementally efficacious. **Trial Registration-** Not Applicable.

**Supplementary Information:**

The online version contains supplementary material available at 10.1186/s12893-023-02135-y.

## Introduction

As one of the most feared postoperative complications, deep vein thrombosis and pulmonary embolisms are collectively known as venous thromboembolism (VTE) [1]. When thromboprophylaxis is implemented properly, it is one of the most common preventable causes of death.

There is a high risk of VTE developing in patients undergoing major abdominal surgery. VTE is also significantly increased in this group of patients when they are immobilized for > 40 min, when they receive anesthesia, or when they use anaesthetic [2, 3]. Subsequently, evidence-based guidelines have been developed to reduce VTE risk, limit inappropriate practice, and improve efficiency. [4–7] Literature suggests, however, that they are not always followed. In hospitalized patients, guideline adherence and interventions for preventing venous thromboembolism are lacking [8, 9, 10].

There are a variety of guidelines and risk stratification tools available in use today, which vary depending on the type of surgery and the health administration network where the surgery is being performed. For major abdominal surgery, including colectomy, the Royal Australasian College of Surgeons (RACS) [6] recommends five to ten days of anticoagulant prophylaxis with heparin or low-molecular-weight heparin (LMWH). As an additional means of prevention, graduated compression stockings (GCS) *and/or* intermittent pneumatic compression devices (IPCD) should be worn  [6]. A recent meta-analysis for the use of IPCD in major abdomainl surgery [11] indicated that IPCDS appear to be efficacious in preventing VTE formation. Their comparative efficacy with respect to other forms of thrombo-prophylaxis is limited and is poorly studied. Their additional efficacy when used in combination with chemical prophylaxis and GCS is also limited although there may be a role for their use as additional therapy when bleeding is a risk factor.

The VTE current guidelines recommend the combined use of chemical prophylaxis, graduated compression stockings (GCS), ***and/or*** intermittent pneumatic compression devices (IPCDs) for venous thromboembolism (VTE) prophylaxis. An audit conducted by the authors at a tertiary hospital confirmed the implementation of all three methods are being used for abdominal surgery outside what is recommended when bleeding is not a risk factor. To gain a comprehensive understanding of the prevailing approach to VTE prophylaxis, a survey was conducted among colorectal and general surgeons involved in abdominal surgery. The study aimed to investigate their attitudes towards VTE prevention, including the utilization of guidelines, the use of risk assessment tools, and variations in perioperative care practices. The ultimate goal was to assess the necessity for future prospective trials on the use of IPCDs and the potential participation of surgeons in such trials.

## Methods

An examination of current VTE prescribing practices in colorectal surgery in Australia and New Zealand was conducted. The survey protocol was approved by the Hunter New England regional ethics review board [Reference number 2018/ETH00331]. Those who participated in the survey did so voluntarily and provided their informed consent.

CSSANZ and GSA both reviewed and approved the survey tool for distribution to their members. In two rounds (September 2019 and then January 2020), members received an email containing a link to an online survey in the Research Electronic Data Capture (REDCapTM) [12, 13] software database. (S1) The survey targeted all registered practicing general and colorectal surgeons, capturing a wide range of knowledge and experience. Questions 1 to 5 of the survey asked about the surgeon’s clinical practice and themselves. For patients undergoing colorectal resections or major abdominal surgery, questions 6–10 asked the surgeon for their opinion and use of clinical practice guidelines for VTE prophylaxis. These questions also determined to what extent surgeons believed intermittent pneumatic compression devices prevented VTE. Finally, we asked surgeons if they would be willing to participate in a large multicenter randomized controlled trial (RCT) on IPCDs for mechanical VTE prevention. It was voluntary to complete the survey, and all data was kept confidential and anonymous. REDCapTM [12, 13] was used to store all data and Microsoft ExcelTM was used to analyze it.

## Results

A total of 214 people responded to the survey, ending this survey with a 10% response rate (2180 particpants). 81% of the respondents were colorectal surgeons from Australia and New Zealand (CSSANZ). The majority of respondents (83%) were male, aged 46–55 years (37%) and from a metropolitan hospital (73%) (Table 1), with 20–50 (26%) being the highest number of colorectal resections performed annually (Table 2). Among surgeons surveyed, 31% used an assessment tool to assess risk. In most cases, respondents who used risk assessment tools used a tool developed by their hospital or state guidelines (25%). There was a lack of popularity and awareness of validated VTE risk assessment tools such as Padua, IMPROVE, NHS and CAPRINI.

**Table 1:** Demographics of respondents

Gender	Male	176	83%
	Female	37	17%
	Undefined	0	
**Age**	25–35	12	5%
	36–45	71	33%
	46–55	80	37%
	56–65	43	20%
	Over 65	7	3%
**Main Work Area**	Metropolitan Hospital	156	73%
	Regional Hospital	43	20%
	Rural Hospital	14	6%

**Table 2:** Rates of colorectal resections among survey respondents

How many colorectal resections per year?		
< 10	43	20%
10–20	30	15%
20–50	56	26%
50–100	41	19%
> 100	43	20%

All respondents used some form of prophylaxis for laparoscopic colectomy/abdominal surgery. Not one respondent would give chemical prophylaxis alone. 54% of surgeons use a combination of all three modalities (chemical plus GCS and IPCDs) only in the operating theatre (OT) and 40% use all three in the pre and post-operative post-operatively. There was not much difference in practice between open and laparoscopic surgery with 51% of surgeons using all three modalities in OT only and 42% pre and post-operatively for open abdominal surgery (Table 3).

**Table 3:** Venous thromboembolism prophylaxis prescribing practices by surgeons for patients undergoing colectomy/major abdominal surgery (N = 214)

What VTE prophylaxis would you usually prescribe for a patient undergoing colectomy/major abdominal surgery?	LAPAROSCOPIC (N/%)	OPEN (N/%)
Chemical only	0	0
Chemical + GCS	5 (2)	7 (3)
Chemical + GCS + IPCDs only in OT	116 (54)	109 (51)
Chemical + GCS + IPCDs intra- and postoperatively	85 (40)	89 (41)
I would not recommend any pharmaceutical VTE prophylaxis	0	0
(Other) Chemical + IPCDs only in OT	7 (3)	8 (4)
Missing data	1 (1)	1 (1)

VTE, venous thromboembolism; GCS, graduated compression stockings; IPCDs, intermittent pneumatic compression devices; OT, operating theatre.

Surgeons were most likely to prescribe VTE prophylaxis based on gender (95%) followed by cardiac risk factors (81%) and cancer (82%). Additionally, smoking (78%), previous VTE (67%), obesity (60%) and age (59%) were risk factors. In high-risk patients having abdominal surgery, 67% of responders would not recommend or prescribe thromboprophylaxis post-discharge.

Forty two percent of respondents had equipoise for a clinical trial on the use of IPCDs for VTE prevention following colectomy and the vast majority (> 95%) felt that IPCDs should provide at least a 2% improvement in clinical VTE rate in order to justify their routine use (Table 4). The rate, type and timing of VTE prophylaxis did not change from the first respondents in the survey to the second respondents, highlighting the fact that this is most likely a representative sample.

**Table 4:** Surgeon opinions regarding intermittent pneumatic compression devices and reduction of venous thromboembolism rates and the use of extended prophylaxis

		Response (N/%)	Surgeons who would participate in an RCT (N/%)
If you use IPCDs, to what extent do you feel their use reduces VTE?	1–3%	50 (23)	27 (13)
	4–6%	67 (31)	31 (14)
	7–9%	19 (9)	8 (4)
	10–12%	33 (16)	16 (7)
	≥ 13%	27 (13)	7 (2)
	N/A	15 (7)	2 (1)
	Missing data	3 (1)	123 (58)
Do you use extended chemical prophylaxis following colectomy/major abdominal surgery?	Yes	70 (33)	
	No	143 (67)	

VTE, venous thromboembolism; IPCDs, intermittent pneumatic compression devices; RCT, randomized controlled trial.

## Discussion

The survey highlights several interesting factors regarding VTE prevention among surgeons. It indicates a high and appropriate use of VTE prophylaxis for colectomy/major abdominal surgery and extensive use of all three forms of prophylaxis (chemical, GCS and IPCDs). The most striking aspect of this survey is the lack of use of risk assessment tools. As a result, this finding stands in stark contrast to the Australian Commission on Safety and Quality in Health Care’s  [7, 8] recommendations for preventing VTE. In their first quality statement, they emphasize the importance of risk assessment; patients potentially at risk (determined by local hospital/unit policies) are assessed for VTE risk using locally endorsed evidence-based tools to determine whether VTE prevention is necessary. “The result should be documented at the time of the assessment, in a place that is easily accessible to all clinicians involved in the patient’s care” [7, 8].

Due to the fact that all surgeons in our survey used VTE prophylaxis, this finding probably does not represent ignorance on their part. This probably represents a ‘blanket’ treatment policy with all patients undergoing these procedures being treated as if they were at high risk and not at risk of bleeding. While this may seem like a defensive approach to a high-risk outcome, it is more likely a more safe approach by experienced clinicians in order to avoid episodes of undertreatment. As a result of this finding, policymakers could use it in the future to prevent costly and sometimes confusing risk assessment tools for patients undergoing high-risk procedures.

Another interesting finding of this survey is the similarity of open vs. laparoscopic VTE prophylaxis. In most cases, surgeons used both forms of surgical access equally, with only a few decreasing the use of IPCDs post-operatively. It may represent an acknowledgment that laparoscopic abdominal surgery still carries a high risk, although this group of patients is more mobile afterwards. Moreover, the survey findings highlight a consistent approach to VTE prevention, where the same prophylactic strategy is applied regardless of changes in individual patient risk factors. This approach aims to prevent undertreatment but may not fully address the evolving needs of patients with varying risk profiles.

There is a clear discrepancy between this survey’s findings and the literature regarding the use of postdischarge prophylaxis. A Cochrane systematic review and meta-analysis of randomized clinical trials for abdominal or pelvic surgery in 2016  [14] concluded that extended prophylaxis with low-molecular-weight heparin decreased asymptomatic venous thromboembolism (5.3% v 13.2%, OR 0.38, 95% CI 0.26 to 0.54). In a more detailed analysis of these results, however, there was no significant reduction in symptomatic VTE (0.1% vs. 1.0%, OR 0.30, 95% CI 0.08 to 1.11). This survey did not examine the reasons for or against extended prophylaxis, but it would appear on the surface that surgeons are aware of the literature, but there remains mixed opinions regarding the importance of continuing VTE prophylaxis given this small, potentially unproven clinical benefit.

There is an intriguing trend in the high utilisation of both mechanical prophylaxis forms. There is a paucity of evidence to suggest that the concurrent use of both GCS and IPCD is superior to either one of these alone. In spite of what might seem intuitive, combination therapy may not be beneficial. The effectiveness of both forms of mechanical prophylaxis with regard to post-operative VTE prevention has been demonstrated, but there is no evidence that one form is superior to the other. Despite the potential benefits of IPCDS, they have a greater risk of pressure related injuries and hinder mobility, which is counterintuitive for preventing VTE. In addition to being costly, they have a significant environmental impact since they are single-use large plastic items. According to the survey, clinicians may underestimate IPCD risks and costs due to the high rate of use.

## Conclusion

There is a clear gap between VTE guidelines and VTE prophylaxis. This indicates a discrepancy between the recommendations provided by established guidelines for preventing venous thromboembolism and the actual practices followed in prescribing VTE prophylaxis. Despite the fact that a majority of surgeons currently utilize IPCDs, the survey results suggest that they are willing to explore the possibility of conducting future trials to assess the effectiveness of IPCDs for VTE prophylaxis. This highlights the crucial need for further research in this area, considering the limited existing evidence.

## Limitations

The poor response rate is indeed a significant limitation when reviewing this surgeon survey. It is challenging to generalize the survey findings to the entire surgeon population. While a low response rate remains a limitation, the consistency in responses from those who responded in the first round to those in the second round of emails, suggests a level of internal consistency and reliability within the sample.

Another limitation is that the question regarding “What VTE prophylaxis would you usually prescribe for a patient undergoing LAPAROSCOPIC colectomy/major abdominal surgery” could have been misinterpreted and this question could have been answered for other major abdominal surgeries. It’s important to note that this question can be applicable to various major abdominal surgeries, not limited to a specific procedure. This issue of a potential gap between guidelines and practice can be a concern across different surgical contexts. Addressing this gap is crucial to ensure consistent and appropriate VTE prophylaxis for patients undergoing major abdominal surgeries.

### Electronic supplementary material

Below is the link to the electronic supplementary material.

Supplementary Material 1

## Data Availability

The datasets used and/or analysed during the current study are available from the corresponding author on reasonable request.

## List of abbreviations

CSSANZ: Colorectal Surgical Society of Australia and New Zealand
DVT: Deep Vein Thrombosis
GCS: Graduated Compression Stockings
GSA: General Surgeons Australia
IPCD: Intermittent Pneumatic Compression Devices
JHH: John Hunter Hospital
LMWH: Low Molecular Weight Heparin
PE: Pulmonary Embolism
RACS: Royal Australasian College of Surgeons
RCT: Randomised Controlled Trial
REDCap: Research Electronic Data Capture
VTE: Venous Thromboembolism

## References

[1] Kakkos SK, Caprini JA, Geroulakos G, Nicolaides AN, Stansby G, Reddy DJ, Ntouvas I (2016). Combined intermittent pneumatic leg compression and pharmacological prophylaxis for prevention of venous thromboembolism. Cochrane Database Syst Rev.

[2] Fan C, Jia L, Fang F, Zhang Y, Faramand A, Chong W, Hai Y (2020). Adjunctive intermittent pneumatic compression in hospitalized patients receiving pharmacologic prophylaxis for venous thromboprophylaxis: a systematic review and meta-analysis. J Nurs Scholarsh.

[3] Gould MK, Garcia DA, Wren SM, Karanicolas PJ, Arcelus JI, Heit JA, Samama CM. (2012). Prevention of VTE in nonorthopedic surgical patients: antithrombotic therapy and Prevention of thrombosis, 9th ed: american college of chest Physicians evidence-based clinical practice guidelines. Chest, 141(2 Suppl), e227S-e277S. 10.1378/chest.11-2297 PMC3278061 22315263

[4] Kakkos SK, Nicolaides AN, Caprini JA (2020). Interpretation of the PREVENT study findings on the adjunctive role of intermittent pneumatic compression to prevent venous thromboembolism. Ann Transl Med.

[5] Clinical Excellence Commission. Prevention of Venous Thromboembolism. (2019) https://www1.health.nsw.gov.au/pds/ActivePDSDocuments/PD2019_057.pdf.

[6] The Australian and New Zealand Working party The Royal Australasian College of Surgeons. : (RACS) (4th Edition). The Australian and New Zealand Working Party on the Management and Prevention of Venous Thromboembolism. https://www.surgeons.org/media/19372/VTE_Guidelines.pdf.

[7] NSW Health. (2019). Prevention of Venous Thromboembolism. Retrieved from https://www1.health.nsw.gov.au/pds/ActivePDSDocuments/PD2019_057.pdf.

[8] Venous Thromboembolism Prevention Clinical Care Standard. Australia: Australian Commission on Safety and Quality in Health Care; 2018.

[9] Watts L, Grant D. Venous thromboembolism (VTE) risk assessment and prophylaxis in acute orthopaedic admissions: improving compliance with national guidelines BMJ Open Quality 2013;2:u202229.w1118. doi: 10.1136/bmjquality.u202229.w1118. 10.1136/bmjquality.u202229.w1118 PMC4663810 26734209

[10] Steiner D, Ay C (2022). Risk assessment and primary prevention of VTE in patients with cancer: advances, challenges, and evidence gaps. Best Pract Res Clin Haematol.

[11] Lott N, Robb F, Nolan E, Attia J, Reeves P, Gani J, Smith S (2022). Efficacy of intermittent compression devices for thromboembolic prophylaxis in major abdominal surgery: a systematic review and meta-analysis. ANZ J Surg.

[12] Harris PA (2019). The REDCap consortium: building an international community of software platform partners. J Biomed Inform.

[13] Harris PA (2009). Research electronic data capture (REDCap)--a metadata-driven methodology and workflow process for providing translational research informatics support. J Biomed Inform.

[14] Felder S, Rasmussen MS, King R, Sklow B, Kwaan M, Madoff R, Jensen C (2019). Prolonged thromboprophylaxis with low molecular weight heparin for abdominal or pelvic surgery. Cochrane Database of Systematic Reviews.

