# Supplementary material for: Venous thromboembolic prophylaxis: current practice of surgeons in Australia and New Zealand for major abdominal surgery
Source: BMC Surg. 2023 Sep 1;23:265. doi: 10.1186/s12893-023-02135-y (PMC10474754; doi:10.1186/s12893-023-02135-y)
Supplement: Supplementary file 1 — Supplementary Material 1 [file 12893_2023_2135_MOESM1_ESM.docx]

**Supporting Information**

**S1-** Questionnaire sent to general and colorectal surgeons in Australia and New Zealand

| Dear Colleagues  Surgical researchers at the John Hunter Hospital are interested in the current practice of colorectal and general surgeons in regards to venous thromboprophylaxis in patients undergoing a colectomy/major abdominal surgery.  By completing this survey, you are indicating your consent to participate to this survey project. If you do not want to participate then close this and delete the email.  Thank you | |
| --- | --- |
| **A. Questions 1- 5 are about you and your clinical practice** | |
| What organisation are you a member of? | - General Surgeons Australia (GSA) - Colorectal Surgical Society Australia and New Zealand (CSSANZ) - GSA and CSSANZ |
| Gender | - Male - Female - Undefined |
| Age | - 25-35 - 36-45 - 46-55 - 56-65 - Over 65 |
| Main Work Area | - Metropolitan Hospital - Regional Hospital - Rural Hospital |
| How many colorectal resections per year? | - < 10 - 10-20 - 20-50 - 50-100 - > 100 |
| Do you use a venous thromboembolism (VTE) risk Yes assessment tool/model? | - Yes - No |
| Which of the following VTE risk assessment tool/models does your facility use? | - Padua VTE tool/model tool/models - IMPROVE VTE tool/model - NHS VTE tool/model - Caprini VTE tool/model - other (please provide name of tool/model) - Not aware of specific risk assessment tool |
| **B. Questions 6-11 are about your opinion and use of clinical practice guidelines for thromboprophylaxis in adult patients having colorectal resection surgery or major abdominal surgery** | |
| What VTE prophylaxis would you usually prescribe for a patient undergoing LAPAROSCOPIC colectomy/major abdominal surgery? | - Chemical only - Chemical + compression stockings + intermittent pneumatic compression devices (IPCD) only in OT - Chemical + compression stockings + IPCDs intra and post-operative post-operatively - I would not recommend any pharmacological VTE prophylaxis - Other - Please specify |
| What thromboprophylaxis would you usually prescribe for a patient undergoing OPEN colectomy/major abdominal surgery? | - Chemical only - Chemical + compression stockings + intermittent pneumatic compression devices (IPCD) only in OT - Chemical + compression stockings + IPCDs intra and post-operative post-operatively - I would not recommend any pharmacological VTE prophylaxis - Other - Please specify |
| If you modify your practice of VTE prophylaxis prescription for the above two operations what is mostly likely to influence your decision? (Tick as many applicable) | - Cancer - Age - Obesity - Gender - Smoking habits - Cardiac risk factors P - Previous Venous- thromboembolism (VTE) |
| Do you regularly use post discharge chemical VTE prophylaxis for patients following colectomy/major abdominal surgery? | - Yes - No |
| If you use IPCD to what extent do you feel their use reduces VTE? | - 1-3%. - 4-6%. - 7-9%. - 10-12%. - >=13%. - Not applicable |
| Would you consider enrolling your patients in a randomised clinical trial assessing the additional use of IPCDs over and above chemical VTE prophylaxis and compression stockings (CS) in patients undergoing major abdominal surgery? (If you have the clinical research resources) | - Yes - No |
